# Serum MOG IgG titres should be performed routinely in the diagnosis and follow-up of MOGAD: Yes

**DOI:** 10.1177/13524585231172954

**Published:** 2023-05-25

**Authors:** Markus Reindl, Kevin Rostasy

**Affiliations:** Clinical Department of Neurology, Medical University of Innsbruck, Innsbruck, Austria; Department of Pediatric Neurology, Witten/Herdecke University, Datteln, Germany

During the last years, myelin oligodendrocyte glycoprotein (MOG) IgG antibody associated disorders (MOGAD), a newly defined entity of acquired demyelinating syndromes, has been recognized as separate disease entity with recently published diagnostic guidelines.^1^ Children and adults with MOGAD present with diverse clinical phenotypes such as monophasic or recurrent presentations of acute disseminated encephalomyelitis (ADEM), optic neuritis, or transverse myelitis, and less commonly with cerebral cortical encephalitis, brainstem–or sole cerebellar presentations. For diagnostic and in particular for prognostic purposes, we recommend that the measurement of MOG-IgG titres should be done routinely both, at onset and follow-up for the following reasons:

## The measurement of serum MOG IgG titres is essential for the diagnosis of MOGAD

In the recently proposed diagnostic criteria we have distinguished clear positive and low positive MOG-IgG results measured by fixed or live cell-based assays (CBA) for the diagnosis of MOGAD.^1^ Both, a clear or low positive MOG-IgG serostatus is defined by quantitative results such as titres or flow cytometry binding ratios. Whereas clear positive MOG-IgG titres are strongly associated with the clinical features proposed for MOGAD (optic neuritis, myelitis, ADEM, cerebral monofocal or polyfocal deficits, brainstem and cerebellar deficits and cortical encephalitis with seizures) and at the same time distinguish them from clinical features characteristic of AQP4-IgG-seropositive neuromyelitis optica spectrum disorder (NMOSD) or multiple sclerosis (MS). In contrast, low titres of MOG-IgG are less specific and are also seen in a small proportion of patients with MS, other neurological diseases, and healthy individuals.^2–5^ The diagnosis of MOGAD can therefore be made based on key clinical features alone if the MOG-IgG result is clearly positive, whereas in the case of a low positive MOG-IgG result additional supporting clinical or magnetic resonance imaging (MRI) features are required. Moreover, recent international multicentre MOG-IgG assay comparison experiments clearly demonstrated that only high titre MOG-IgG results, but not low-titre MOG-IgG results, are comparable and reproducible among individual diagnostic centres.^3,6^ Thus, both the positive predictive value and the reproducibility of MOG-IgG results depends on quantitative values which clearly demands for the routine measurement of titres.

## The measurement of serum MOG IgG titres is important to assess the risk for a relapse in MOGAD

The majority of children and adults with an initial MOGAD episode will have a monophasic disease course but depending on the study up to 40% will have further episodes.^1^ At present it is not possible to predict who will have a relapsing disease course overtime. Neither MOGAD subtype, MRI features or clinical severity are helpful for predicting the future disease course. Likewise, onset serum MOG-IgG titres, despite being important for the diagnosis of MOGAD, do not predict recovery or relapse.^1,7^ In most patients MOG-IgG titres decline with time, but may also remain positive in other patients for years. Several studies found that persistent MOG-IgG seropositivity is associated with an increased likelihood of having a relapse by a factor of 2–10^7–10^ particularly when the MOG-IgG titre remains high.^7,9,10^

In a recent study of 116 children with MOGAD, MOG-IgG titres decreased significantly overtime in patients with a monophasic disease course compared to patients with a relapsing disease course in the majority of children.^9^ This decline of MOG-IgG titres was most prominent during the first 12 months after disease onset and a seroconversion defined as a MOG-IgG titre of less than 1:160 during the first 24 months was shown to have a high positive predictive value for a monophasic disease course. Interestingly, no patient in our cohort suffered from a relapse after time of first seroconversion, although fluctuating MOG-IgG titres with increase after a period of low or even negative MOG-IgG titres were reported in selected patients. This study shows that a seroconversion to negative MOG-IgG titres is associated with a significantly reduced risk for further relapses in paediatric MOGAD. In 70% of patients with monophasic MOGAD, titres decreased to negative levels during follow-up, whereas 93% of patients with polyphasic MOGAD still had elevated MOG-IgG titres at last follow-up.

Similar results were reported in another study investigating 102 paediatric and adult patients with MOGAD.^10^ Lower MOG-IgG titres at remission or seroconversion to negative was associated with a significantly reduced risk for further relapses. Thus, both studies provide strong evidence that a decrease of MOG-IgG titres within the first 2 years after onset is associated with a reduced risk for a relapsing diseases course. Therefore, we suggest that longitudinal MOG-IgG titres should be routinely assessed in MOGAD patients.

## Conclusion

At present it is not possible at disease onset to predict the future course of a patient with MOGAD. But knowledge of persisting or in particular declining MOG IgG titres overtime is important because it has been shown to be associated with a more favourable disease course in the latter. Therefore, we recommend that serum MOG IgG titres should be performed routinely in the diagnosis and follow-up of MOGAD.
